# Small Object Detection in Traffic Scenes Based on YOLO-MXANet

**DOI:** 10.3390/s21217422

**Published:** 2021-11-08

**Authors:** Xiaowei He, Rao Cheng, Zhonglong Zheng, Zeji Wang

**Affiliations:** College of Mathematics and Computer Science, Zhejiang Normal University, Jinhua 321004, China; chengrao@zjnu.edu.cn (R.C.); zhonglong@zjnu.edu.cn (Z.Z.); wangzj@zjnu.edu.cn (Z.W.)

**Keywords:** deep learning, computer vision, intelligence transportation, YOLOv3, lightweight

## Abstract

In terms of small objects in traffic scenes, general object detection algorithms have low detection accuracy, high model complexity, and slow detection speed. To solve the above problems, an improved algorithm (named YOLO-MXANet) is proposed in this paper. Complete-Intersection over Union (CIoU) is utilized to improve loss function for promoting the positioning accuracy of the small object. In order to reduce the complexity of the model, we present a lightweight yet powerful backbone network (named SA-MobileNeXt) that incorporates channel and spatial attention. Our approach can extract expressive features more effectively by applying the Shuffle Channel and Spatial Attention (SCSA) module into the SandGlass Block (SGBlock) module while increasing the parameters by a small number. In addition, the data enhancement method combining Mosaic and Mixup is employed to improve the robustness of the training model. The Multi-scale Feature Enhancement Fusion (MFEF) network is proposed to fuse the extracted features better. In addition, the SiLU activation function is utilized to optimize the Convolution-Batchnorm-Leaky ReLU (CBL) module and the SGBlock module to accelerate the convergence of the model. The ablation experiments on the KITTI dataset show that each improved method is effective. The improved algorithm reduces the complexity and detection speed of the model while improving the object detection accuracy. The comparative experiments on the KITTY dataset and CCTSDB dataset with other algorithms show that our algorithm also has certain advantages.

## 1. Introduction

Object detection is an essential field of computer vision, and its task is to locate and classify objects with the variable number in an image. Object detection in traffic scenes is an essential part of driverless technology, which adopts image processing or deep learning to detect and identify vehicles, pedestrians, and traffic signs in traffic scenes to lay a good foundation for developing intelligent transportation. Object detection algorithms based on convolutional neural networks are mainly divided into two categories: one is the two-stage algorithms represented by RCNN series [1,2,3], and the other is the one-stage algorithms represented by SSD series [4,5] and YOLO series [6,7,8]. Object detection algorithms based on anchor-free [9,10,11,12] are developing rapidly in the one-stage algorithms. Two-stage algorithms depend on the proposals, and their detection speed is generally slow, in other words, their real-time performance cannot meet the demand of traffic scenes, even though its detection accuracy is constantly improving. The speed of one-stage algorithms based on regression is fast enough to satisfy the requirements of most tasks. However, there is still room for improvement in detection accuracy. At present, many scholars have applied general object detection algorithms to the traffic field. Que Luying et al. [13] proposed a lightweight pedestrian detection engine with a two-stage low-complexity detection network and adaptive region focusing technique, which not only reduced the computational complexity but also maintained sufficient detection accuracy. Yang Xiaoting et al. [14] proposed a novel scale-sensitive feature reassembly network (SSNet) for pedestrian detection in road scenes. Ma Li et al. [15] studied and solved the problem that YOLOv3-tiny has a high missed detection rate for small-scale objects such as pedestrians in real-time detection; however, the accuracy of their algorithm cannot satisfy the requirements in actual scenes. Guo Fan et al. [16] proposed the traffic sign detection network (YOLOv3-A) based on an attention mechanism to solve the misdetection and omission of small objects. Liu Changyuan et al. [17] proposed the vehicle target detection network (YOLOV3-M2), which promoted the detection efficiency and enhanced the detection ability of small targets; however, it only detected a single class of targets.

In object detection, when the size of an object is small enough relative to the size of the original image, we usually consider the object as a small object. For small objects, some datasets have a clear definition. For example, CityPerson, the pedestrian dataset, defines objects less than 75 px high as small objects in the raw image with the size of 1024×2048. In the MS COCO dataset, small objects are the objects with pixels less than 32×32. In the traffic sign dataset, Zhu et al. [18] defined the objects whose width accounted for less than 20% of the whole image as small objects. The current general object detection algorithms have achieved a good detection effect for large and medium objects. However, because of the smaller coverage area, lower resolution, weaker feature expression ability, and little feature information of small objects, the above general object algorithms are not good at detecting small objects. Recently, many researchers have focused their attention on small object detection. Wang Hongfeng et al. [19] proposed a generative adversarial network (GAN) capable of image super-resolution and two-stage small object detection, which exhibited a better detection performance than mainstream methods. Bosquet Brais et al. [20] introduced STDnet-ST, an end-to-end spatiotemporal convolutional neural network for small object detection in video, which achieved state-of-the-art results for small objects. Lian Jing et al. [21] proposed a small object detection method in traffic scenes based on attention feature fusion, which improved the detection accuracy of small objects in traffic scenes. Zhang Can et al. [22] proposed a neural network for detecting small objects based on original Cascade RCNN, which performed better not only in small object detection but also in industrial applications.

In general, there are multiple objects, small objects, and occluded objects in complex traffic scenes [23], and it is difficult for traditional object detection methods to obtain better detection results. Therefore, it is necessary to study more algorithms of small object detection in traffic scenes. In recent years, the continuous improvement of network performance has led to the increase of model size and computation. With the popularity of mobile embedded devices, the deep neural network can be better applied to mobile devices only when the precision, parameter size, and inference speed are well balanced. Deploying well-performing algorithms on mobile devices is a trend. For example, Chen Rung-Ching et al. [24] developed a real-time monitoring system for home pets using raspberry pie. In our paper, to reduce the amount of calculation and the number of parameters while maintaining a better detection accuracy and speed, YOLO-MXANet is proposed by using the YOLOv3 algorithm for reference. CIoU [25] is adopted to improve the loss function, which makes the bounding box regress better. Although the lightweight network MobileNeXt [26] dramatically reduces the number of parameters and computational effort by using depthwise separable convolution, it has weaker feature extraction capability. To improve the feature extraction capability of MobileNeXt, the Shuffle Channel and Spatial Attention (SCSA) module is embedded into the SGBlock module, which can model long-distance dependency well to highlight the features of small objects. For the dataset, Mosaic [27] and Mixup [28] are used to enhance the robustness of the model. In the process of feature fusion, the Multi-scale Feature Enhancement Fusion (MFEF) network is proposed, in which an additional Down-top path is added, and the four-fold subsampled feature maps are fused to extract the features of small objects effectively. Meanwhile, the idea of CSPNet [29] is utilized to combine the convolution operation to reduce the number of network parameters and amount of calculation. In our work, the SiLU activation function is adopted into the Convolution-Batchnorm-SiLU (CBS) and A-SGBlock module to accelerate the convergence of the model. The experimental results on KITTI and CCTSDB datasets show that YOLO-MXANet in this paper has lower computational complexity and smaller number of parameters while improving the detection accuracy and speed. Compared with the original YOLOv3, the detection performance of the model is greatly enhanced while the speed is promoted, and the complexity of the model is lower. Compared with the latest algorithms, YOLO-MXANet also has certain advantages in detection accuracy and model complexity.

## 2. Baseline and YOLO-MXANet Algorithm

In this section, firstly, YOLOv3 baseline algorithm will be introduced in Section 2.1. Then, in Section 2.2, our proposed algorithm will be organized through five sub-sections. In Section 2.2.1, the backbone network SA-MobileNeXt will be presented and explained. In Section 2.2.2, Multi-scale Feature Enhancement Fusion Network will be elaborated. In Section 2.2.3, SiLU activation Function will be described in detail. In Section 2.2.4, the data enhancement approach utilized will be explained. In Section 2.2.5, the loss function used will be presented.

### 2.1. YOLOv3 Baseline Algorithm

YOLOv3 uses the Darknet-53 backbone network to extract features, which integrates the residual idea of ResNet [30]. The advantage of residual structure in the Darknet-53 (named Res_Unit) is that the accuracy can be improved by increasing the depth of network. The Res_Unit block uses the shortcut, which can alleviate the gradient diffusion problem caused by increasing the depth of the network. In addition, YOLOv3 utilizes three different feature layers extracted from the Darknet-53 backbone network to fuse and form three prediction layers for prediction. In the YOLOv3, the feature fusion idea of FPN [31] is adopted. That is, the semantic information and location information of three feature maps with different scales are combined by up-sampling and fusion to obtain feature maps containing rich information for detection. Therefore, YOLOv3 can effectively detect small objects. Specifically, the image with the size of 640×640 is sent into the network, and three feature maps with different scales (e.g., 80×80,40×40,20×20) are obtained. The 32-fold downsampled feature maps from the backbone network pass through five convolution layers. On the one hand, the feature maps generated are directly predicted after passing through one convolution layer. On the other hand, after a convolution layer and an upsampling operation, they are concatenated with the 16-fold downsampled feature maps from the backbone network to obtain the fusion feature maps. The operations of the 16-fold downsampled feature maps from the backbone network are similar to those of the 32-fold downsampled feature maps.

YOLOv3 employs the K-means algorithm to determine the size of the prior box. Although too many prior boxes can guarantee the effect, it greatly affects the detection speed of the model, so it gets nine prior boxes by clustering on the COCO dataset. The feature maps with a single scale utilize three prior boxes, and the corresponding relationship between prior boxes and feature maps with different scales is as follows. In detail, the 32-fold downsampled feature maps use the following three prior boxes: [(116,90); (159,198); (373,326)]; the 16-fold downsampled feature maps apply the following three prior boxes: [(30,61); (62,45); (59,119)]; the 8-fold downsampled feature maps employ the following three prior boxes: [(10,13); (16,30); (33,23)]. Large feature maps with small receptive fields are very sensitive to small-scale objects, so small prior boxes are selected. On the contrary, small feature maps with large receptive fields are suitable for detecting large objects, so large prior boxes are selected.

### 2.2. YOLO-MXANet Algorithm

#### 2.2.1. SA-MobileNeXt

Although numerous residual modules can extract sufficient feature information, the Darknet-53 has numerous parameters and demands a large amount of computation. The deployment of convolutional neural networks on embedded devices is challenging due to the limited memory and computing resources. In order to balance the complexity, the detection speed, and the detection accuracy of the model, in this paper, we propose the lightweight feature enhancement backbone network called SA-MobileNeXt.

**Figure 1:**
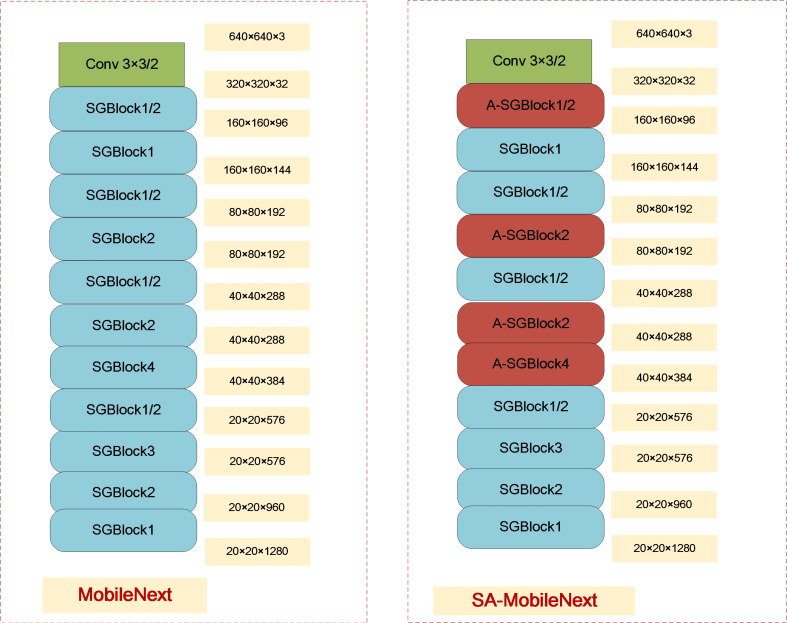
The structure of MobileNeXt and SA-MobileNeXt.

**Figure 2:**
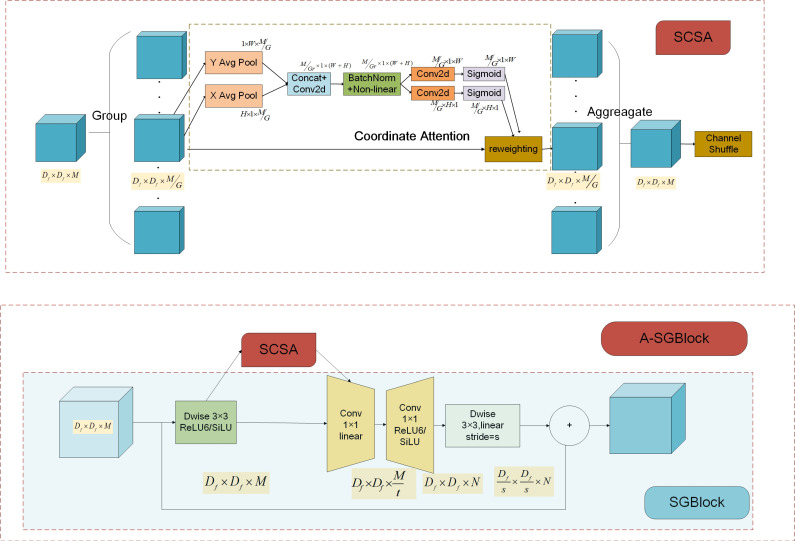
The structure of SGBlock and A-SGBlock. The light blue box represents the specific structure of SGBlock. Based on the SGBlock, A-SGBlock incorporates the SCSA module. SCSA represents the Shuffle Channel and Spatial Attention module. Coordinate Attention includes channel and spatial attention.

To reduce the number of parameters and computation amount of the network, we chose the lightweight backbone network called MobileNeXt as the basic model for improvement to simplify the network model. In recent years, artificially designed lightweight backbone networks have become popular, such as MobileNet Series (e.g., MobileNetv1 [32], MobileNetv2 [33], and MobileNetv3 [34]), ShuffleNet Families (e.g., ShuffleNetv1 [35] and ShuffleNetv2 [36]), and SqueezeNet [37]. The above manually designed backbone networks are built by stacking basic modules. In our work, firstly, the newly proposed lightweight backbone network-MobileNeXt [26] is utilized, which is made up of stacked SandGlass blocks (SGBlock), and its structure is shown on the left of Figure 1. Many studies have proved that the SGBlock is better than the Inverted Residual (IR) blocks in MobileNetv2 to preserve adequate feature information and promote gradient propagation. The specific structure of SGBlock is shown in the light blue box of Figure 2 (*t* represents the reduction rate of dimension, and *s* represents the stride). In detail, two depthwise convolutions are placed at the end of the block, and two pointwise convolutions are placed in the middle of the block. The point convolution can be used to encode the information of internal channels but cannot capture spatial information, and the depthwise convolution can learn more expressive spatial context information. It is worth noting that the first depthwise convolution and the last point convolution utilize the ReLU6 activation function; the first point convolution and the second depthwise convolution directly perform linear output to reduce information loss, and there is no identity mapping in the SGBlock when the input and output channels are different. Mathematically, let $F\in {ℜ}^{D_{f}\times }{}^{D_{f}\times M}$ be the input tensor, and $G\in {ℜ}^{D_{f}\times }{}^{D_{f}\times M}$ be the output tensor of the SGBlock, and the SGBlock can be formulated as follows:

(1) $$\begin{array}{l} \overset{\wedge }{G}=T_{1,p}T_{1,d}(F),\\ G=T_{2,d}T_{2,p}(\overset{\wedge }{G})+F \end{array}$$

where $T_{i,p}$ and $T_{i,d}$ are the *i*-th pointwise convolution and depthwise convolution, respectively. The depthwise separable convolution is used in the SGBlock. Compared with the standard convolution, the depthwise separable convolution includes the depthwise convolution and the point convolution. Assume that the size of the input feature maps is $D_{f}\times D_{f}\times M$, the size of the output feature maps is $D_{f}\times D_{f}\times N$, and the size of standard convolution kernel is $D_{k}\times D_{k}\times M$. The computational cost of the standard convolution is $D_{k}\cdot D_{k}\cdot M\cdot N\cdot D_{f}\cdot D_{f}$, and the computational cost of depthwise separable convolution is $D_{k}\cdot D_{k}\cdot M\cdot D_{f}\cdot D_{f}+M\cdot N\cdot D_{f}\cdot D_{f}$. From the above formulas, we can see that the calculation amount of depthwise separable convolution is much less than the calculation amount of standard convolution [32]. In order to enable the MobileNeXt to be used as the backbone network of YOLOv3, the original MobileNeXt is improved by removing the 7×7 average pooling layer and the fully connected layer to form the backbone network MobileNeXt used in this paper.

Although the lightweight backbone network called MobileNeXt can reduce the amount of computation and the number of parameters in the network, its feature extraction capability is insufficient. The attention module is embedded into the convolutional neural network, which enables the lightweight convolutional neural network to calculate the correlation coefficient of the internal feature points, thus enhancing the internal correlation of the feature maps. Recent studies have found that channel attention (e.g., Squeeze-and-Excitation Attention [38]) is a significant factor in improving the performance of the model. However, they usually ignore the position information. In order to encode more useful position information, the Coordinate Attention (CA) [39] embeds position information into channel attention so that the model could locate the object area more accurately. Although the Coordinate Attention can effectively encode channel and spatial features of small objects, its number of parameters is more than most attention mechanism modules. Therefore, in this paper, the number of parameters of the Coordinate Attention is reduced by grouping features, and the Shuffle Channel and Spatial Attention (SCSA) module is presented, which has fewer parameters than that of the Coordinate Attention module. The embedded position and structure of the SCSA module are shown in Figure 2, and an SGBlock embedded with an SCSA module is called an A-SGBlock, and it can be formulated as follows:

(2) $$\begin{array}{l} \overset{\wedge }{G^{\prime }}=T_{1,p}SCSA(T_{1,d}(F)),\\ G^{\prime }=T_{2,d}T_{2,p}(\overset{\wedge }{G^{\prime }})+F \end{array}$$

where *SCSA* is the attention module, and whose specific operations are described as follows. Firstly, the input feature maps $X\in R^{D_{f}\times }{}^{D_{f}\times M}$ are divided into *G* groups along the channel dimension, i.e., X=[X1,…,XG],Xk∈ℜDf×Df×MG. Secondly, the Coordinate Attention (that is, channel and spatial attention) is performed for each group $X_{k}$, in which the Coordinate Attention decomposes the channel attention into two one-dimensional feature coding processes that aggregates features along with different directions. The advantage of this process is to capture long-range dependencies along one spatial direction and retain accurate position information along the other spatial direction. Thirdly, each group of feature maps that pass through the Coordinate Attention module are fused. Fourthly, the Shuffle Channel [36] promotes information communication between different groups of features.

Precisely, the Coordinate Attention module consists of two steps: coordinate information embedding and coordinate attention generation. Firstly, each channel is encoded along with the horizontal and vertical coordinates by using pooling kernels with sizes (*H*, 1) and (1, *W*), respectively. Mathematically, the output of the *m*-th channel at height *h* and the output of the *m*-th channel at width *w* can be respectively formulated as follows:

(3) $$\begin{array}{l} Z_{m}^{h}\left(h\right)=\frac{1}{W}\sum_{0\leq i<W}x_{m}(h,i)\\ Z_{m}^{w}\left(w\right)=\frac{1}{H}\sum_{0\leq i<H}x_{m}(j,w) \end{array}$$

A pair of direction-aware and position-sensitive feature maps are obtained. Then generated feature maps are fused in spatial dimensions and fed into a shared 1×1 convolution transformation function *T*, and this process can be formulated as follows:

(4) $$f=\delta (T_{1}(z^{h},z^{w}))$$

where [.,.] represents the concatenation operation along the spatial dimension, δ is a non-linear activation function, f∈ℜMGr×H+W is the intermediate feature map, and *r* is the reduction rate to control the module size. Next, the feature maps f obtained in the previous step are divided into two separate tensors fh∈ℜMGr×H and fw∈ℜMGr×W along the spatial dimension. In the next step, two convolution transformation functions $T_{h}$ and $T_{w}$ are used to transform the channel number of feature maps to make it consistent with the channel number of the input feature maps, and this process can be formulated as follows:

(5) $$\begin{array}{l} g^{h}=\sigma (T_{h}(f^{h})),\\ g^{w}=\sigma (T_{w}(f^{w})) \end{array}$$

where σ is the sigmoid activation function. Finally, the input feature maps are multiplied with a pair of feature maps obtained through the steps of coordinate information embedding and coordinate attention generation, and then, attention feature maps are generated to enhance the representation of the region of interest, and this process can be formulated as follows:

(6) $$y_{m}(i,j)=x_{m}(i,j)\times g_{m}^{h}(i)\times g_{m}^{w}(j)$$

Therefore, in order to enhance the ability of lightweight backbone network, in this paper, we present the feature enhancement backbone network called SA-MobileNeXt, which is based on attention and is shown on the right of Figure 1. “SGBlockn/A-SGBlockn” represents “n SGBlock/A-SGBlock modules are used”; if “/2”, it represents “the stride of SGBlock/A-SGBlock is 2”, otherwise it represents “the stride of SGBlock/A-SGBlock is 1”. The SA-MobileNeXt uses the A-SGBlock module in the front part of the backbone network, which embeds the Shuffle Channel and Spatial Attention (SCSA) module proposed in this paper into the SGBlock. In this work, nine A-SGBlock modules are employed for two reasons. On the one hand, using lots of A-SGBlock modules (especially the A-SGBlock modules with numerous channels located at the back of the backbone network) can increase the number of parameters and computation amount, resulting in a decrease in speed while not improving the accuracy. On the other hand, using A-SGBlock modules in the shallow layer of the backbone network can encode more accurate location information, which is conducive to detecting small objects. In addition, in our SA-MobileNeXt, the ReLU6 activation function used in the original SGBlock modules is replaced with the SiLU activation function, making the model converge faster. The SiLU activation function is described in Section 2.2.3.

#### 2.2.2. Multi-Scale Feature Enhancement Fusion Network

**Figure 3:**
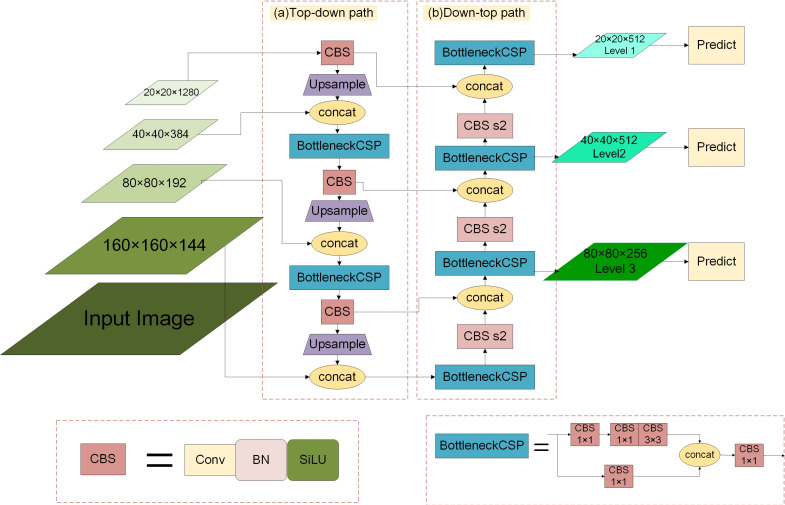
The structure diagram of Multi-scale Feature Enhancement Fusion network. CBS represents the convolution module with stride 1. CBS s2 represents the convolution module with stride 2. BottleneckCSP (BC) represents the combination of convolution module.

In the process of feature fusion, to better integrate the features extracted from the backbone network, the Multi-scale Feature Enhancement Fusion Network is proposed, which further promotes the performance of small object detection. Its main structure is shown in Figure 3 and is explained as follows.

In the original YOLOv3, the feature fusion method of FPN only integrates 8-fold downsampled feature maps, 16-fold downsampled feature maps, and 32-fold downsampled feature maps. However, the shallow features extracted by the backbone are essential for detecting small objects. As a result, the 4-fold downsampled feature maps from the backbone network are integrated to promote small object detection, and the specific operations of fusion are as follows. Firstly, the 8-fold downsampled fusion feature maps with low-resolution pass through a BottleneckCSP and a CBS module and then are processed by an upsampling operation. Finally, the resulting feature maps are fused with feature maps with the size of 160×160×144 from the backbone network.

Meanwhile, the feature fusion method in PANet [40] can better preserve the shallow feature information, a Down-top path (Figure 3b) is added by referring to the method in PANet. We take the fusion process of 8-fold downsampled feature maps in the Down-top path as an example, and its operations are detailed as follows. The 4-fold downsampled fusion feature maps pass through a BottleneckCSP module and then are upsampled by a CBS module with stride 2 to become 8-fold downsampled feature maps, and the resulting feature maps are fused with feature maps with the same resolution from the Top-down path. In order to save the number of parameters and make the model converge faster, we still utilize the last three detection for detection.

The introduction of the 4-fold downsampled feature maps in the backbone network and an additional Down-top path can improve the accuracy of object detection but increase the number of parameters to a certain extent. Therefore, the previous convolution blocks are combined into the BottleneckCSP module by using the idea of CSPNet to further reduce the number of parameters and computation amount in the network without affecting the detection accuracy. The structure of the BottleneckCSP module is shown in the bottom right of Figure 3, and it contains two branches. Firstly, in the first branch, there are three convolution layers. In the second branch, there is a 1×1 convolution layer. Then, the feature maps of the two branches are fused, and finally, the number of channels is transformed by a 1×1 convolution layer. In addition, the CBL modules in the feature fusion network are replaced with the CBS modules, whose structures are shown in the bottom left of Figure 3. As we can see, the optimized CBS modules in this paper use the SiLU (Sigmoid Weighted Linear Unit) to replace the Leaky ReLU.

#### 2.2.3. SiLU Activation Function

**Figure 4:**
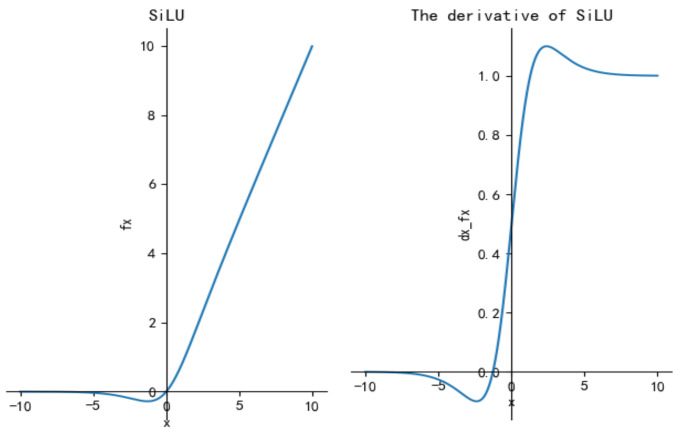
The activation function and derivative curves of SiLU.

In this paper, the optimized SGBlock modules use the SiLU [41] (Sigmoid Weighted Linear Unit) to replace the ReLU6. Meanwhile, the CBS modules utilize the SiLU to replace the Leaky ReLU. The calculation formulas of *SiLU* and its first derivative are as shown in Equation (7) and Figure 4.

(7) $$\begin{array}{l} SiLU=x\cdot sigmoid(x)\\ sigmoid(z)=\frac{1}{1+e^{-z}}\\ {SiLU}^{\prime }=SiLU+sigmoid(1-SiLU) \end{array}$$

If the input value is greater than 0, the *SiLU* is approximately the same as the ReLU; and if the input value is less than 0, the value of *SiLU* approaches 0. Compared with the Sigmoid and Tanh, the *SiLU* activation function does not increase monotonously and has a global minimum value of about -0.28. In general, deep convolutional neural networks often encounter the phenomenon of gradient explosion. However, an attractive feature of SiLU is self-stability: when the derivative is zero, the global minimum can play the role of “soft bottom”, which can inhibit the update of large weights from avoiding gradient explosion.

#### 2.2.4. Data Enhancement

In deep learning, it is crucial to keep the number of samples be sufficient. Numerous samples will make the trained model have a better effect and generalization ability. However, for the KITTI and CCTSDB datasets used in this paper, their sample quantity and quality are not good enough, which will lead to overfitting. Recently, Dewi, Christine et al. [42] combined synthetic images with original images to enhance datasets and verify the effectiveness of synthetic datasets. Therefore, data enhancement is an effective solution to improve the quality of datasets, which can reduce the overfitting phenomenon of the network. A network with better generalization ability can be obtained by transforming the training images, which can better adapt to the application scenarios. Therefore, two methods of data enhancement, Mosaic and Mixup, are adopted in this paper to improve the quality of the dataset so that the proposed improved algorithm is more suitable for training on a single GPU.

The two types of data enhancement are described in detail below. The Mixup merges the positive and the negative samples into a new group of samples, which doubles the size of the sample. Meanwhile, the objects in each batch after Mixup will be more than the objects in the original batch. The Mosaic combines four training images into one in a certain proportion, enabling the model to learn to recognize smaller objects, which can enrich the background of detecting objects and calculate four kinds of images in Batch Normalization, and the batch size does not need to be large so that a GPU can achieve better results.

In this paper, due to the limitation of GPU and model size, and in order to make a fair comparison between different models, the training batch size is uniformly set as 4. We adopted such a data enhancement strategy that uses only the Mosaic data enhancement strategy in the three batches and uses a combination of the Mosaic and Mixup data enhancement strategy in the one batch. Through the experiments, the model trained by our data enhancement strategy is better than the model trained by the Mosaic only in the four batches.

#### 2.2.5. Loss Function

The total loss function used by the YOLO-MXANet algorithm is shown in Equation (8). *CIoU* regression loss is employed to improve MSE regression loss [43], and the improved loss function is more suitable for detecting small objects in traffic scenes. *CIoU* inherits the advantages of Generalized Intersection Over Union (GIoU) [44] and Distance-IoU (DIoU) [45], which not only considers the distance and overlap ratio but also considers the scale and the aspect ratio between the prediction box and the ground truth box so that it can carry out the bounding box regression better [43]. It consists of three parts: the first is *loss_CIoU_*, which represents regression loss; The second part is *loss_obj_*, which represents confidence loss. The third part *loss_class_* represents classification loss.

(8) $$\begin{array}{c} LOSS=loss_{CIoU}+loss_{obj}+loss_{class}\\ loss_{CIoU}=1-CIoU,CIoU=IoU-\frac{\rho^{2}\left(b,b^{gt}\right)}{c^{2}}-\alpha \nu \\ loss_{obj}=-\sum_{i=0}^{K\times K}\sum_{j=0}^{M}I_{ij}^{obj}\left[\overset{\wedge }{C_{i}}\log(C_{i})+(1-\overset{\wedge }{C_{i}})\log(1-C_{i})\right]\\ -\lambda_{noobj}\sum_{i=0}^{K\times K}\sum_{j=0}^{M}I_{ij}^{noobj}\left[\overset{\wedge }{C_{i}}\log(C_{i})+(1-\overset{\wedge }{C_{i}})\log(1-C_{i})\right]\\ loss_{class}=-\sum_{i=0}^{K\times K}I_{ij}^{obj}\sum_{c\in classes}\left[\overset{\wedge }{p_{i}}(c)\log(p_{i}(c))+(1-\overset{\wedge }{p_{i}}(c))\log(1-p_{i}(c))\right] \end{array}$$

## 3. Experimental Results and Analysis

In order to verify the performance of YOLO-MXANet, comparative experiments on the KITTI dataset and CCTSDB dataset are conducted. In this paper, the experimental platforms are Intel^®^ Core™ i7-9700 CPU @ 3.00 GHz processor and NVIDIA GeForce RTX 2080Ti GPU. The algorithms in this paper are programmed in Python 3.8 and implemented in PyCharm Community 2020.2.3 software. To ensure the fairness of test, all models are trained from scratch and trained 200 epochs. To make the training process more stable, the Adam optimizer is used for training. In the training process, the warm-up strategy is adopted in the first three epochs, and the cosine annealing strategy is adopted for training from the fourth epoch to the 200th epoch, which reduces the learning rate from 0.01 to 0.002. The value of Momentum is set to 0.937, and the value of Weight_decay is set to 0.0005.

The following evaluation indexes are used to evaluate the performance of algorithms. The accuracy of detection algorithms is measured by using the Precision, Recall, and F1 score (the harmonic mean value of Precision and Recall). The Average Precision (AP) is used to measure the detection accuracy of each type of object. The mean Average Precision (mAP) is used to measure the average detection accuracy of multi-class objects. The higher the mAP value is, the higher the comprehensive performance of the model in all categories will be. The speed of each image on the GPU is used to measure the detection speed of object detector. The number of parameters and computation amount are used to measure the complexity of the model.

### 3.1. Ablation Learning on the KITTI Dataset

In order to prove the effectiveness of each improvement method, we conduct ablation experiments on the KITTI dataset, and the results are shown in Table 1. The KITTI dataset is randomly and automatically divided into train set, validation set and test set, and the 8:1:1 ratio is adopted in this study. At the same time, eight classes of objects in the dataset are fused into three types of objects, namely Pedestrian, Car, and Cyclist. In our experiment, we use images with the size of 640×640×3 for training and testing. We employ original YOLOv3 as our Scheme A. We first established a more robust YOLOv3 baseline, which has a good performance in terms of speed. Meanwhile, YOLOv3 also has a higher detection accuracy, but the number of parameters and computation amount are large. Based on Scheme A, Scheme B adopts CIoU loss function, which can improve the positioning ability of small objects. Based on Scheme B, Scheme C uses MobileNeXt, which causes a slight reduction in detection performance but simplifies the model by reducing the number of network parameters from 61,508,200 to 22,927,784. At the same time, the detection speed of each image is improved from 3.5 ms to 2.4 ms. Based on Scheme C, Scheme D utilizes Mosaic and Mixup to promote the quality of dataset, which makes up for the performance loss caused by the lightweight network while keeping the speed unchanged and improving the detection ability of small object and the generalization of the network, increasing F1 from 0.812 to 0.842 and mAP 0.5 from 0.865 to 0.897. Based on Scheme D, Scheme E introduces the feature fusion method of PANet and integrates the four-fold subsampled feature maps containing small object information to improve the detection ability of small objects. At the same time, the idea of CSPNet is used to combine convolution blocks, which reduces the number of parameters. From the experimental results, Scheme E improves the detection performance of small objects, which reduces the number of parameters from 22,927,784 to 13,870,888 and increases F1 from 0.842 to 0.861 and mAP 0.5 from 0.897 to 0.905. Based on Scheme E, we introduce the SiLU activation function to make the model converge faster and improve the stability of the model, which replaces original activation function of CBL and SGBlock module with SiLU and makes it become our Scheme F, which increases F1 from 0.861 to 0.877, mAP 0.5 from 0.905 to 0.916. Based on Scheme F, Scheme G introduces the Coordinate Attention mechanism to obtain the valuable features of small objects, which increases mAP 0.5 from 0.916 to 0.922. Based on Scheme G, Scheme H proposes the Shuffle Channel and Spatial Attention (SCSA) module to improve the detection accuracy while further simplifying the model, which not only increases F1 from 0.876 to 0.885 and mAP 0.5 from 0.922 to 0.924 but also decreases the number of parameters from 13,987,271 to 13,874,564. In conclusion, compared with YOLOv3 baseline, our final scheme reduces the number of parameters from 61,508,200 to 13,874,564, and the GFLOPS from 154.9 to 37.0, increasing the speed by 0.6 ms. Meanwhile, the detection performance is improved, which increases F1 by 4.8 percentage points and mAP 0.5 by 3.6 percentage points.

**Figure 5:**
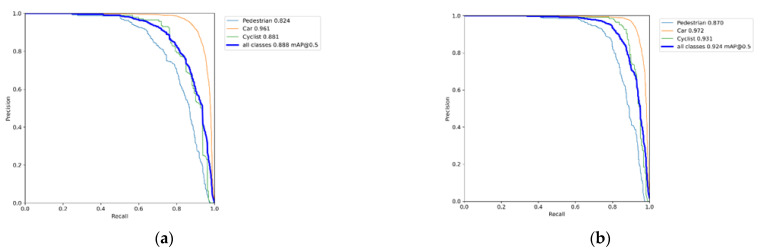
The PR curve diagram of YOLOv3 and YOLO-MXANet on the KITTI dataset. (**a**) YOLOv3. (**b**) YOLO-MXANet.

In order to further prove the excellent effect of improved algorithm, we show the PR curve diagram of YOLOv3 and YOLO-MXANet, as well as the AP value of each category and the mAP value of all categories, which are shown in Figure 5. Compared with YOLOv3, the mAP value of YOLO-MXANet increases by 3.6 percentage points, and the AP value of each category of YOLO-MXANet has increased. Specifically, the AP value of category “Pedestrian” increases by 4.6 percentage points, the AP value of category “Car” increases by 1.1 percentage points, and the AP value of category “Cyclist” increases by 5 percentage points.

### 3.2. Comparison Experiments with Other Algorithms on the KITTI Dataset

In order to further verify the performance of improved algorithm, comparative experiments are conducted between YOLO-MXANet and other algorithms on the KITTI dataset, and the comparison results are shown in Table 2. The experimental results show that YOLO-MXANet has higher detection accuracy compared with YOLOv5s, and YOLO-MXANet has less parameters than YOLOv5m while keeping slightly better accuracy. Compared with YOLOv3 and YOLOv3-SPP, YOLO-MXANet has fewer parameters and more significant advantages in detection accuracy. Compared with the lightweight algorithm YOLOv3-tiny, although the number of parameters of YOLO-MXANet is a little more than that of YOLOv3-tiny, the mAP 0.5 value of YOLO-MXANet is 23.2 percentage points higher than that of YOLOv3-tiny, while the F1 value of YOLO-MXANet is 21.5 percentage points higher than that of YOLOv3-tiny. Compared with the latest lightweight algorithm YOLOv4-tiny, the mAP 0.5 value of YOLO-MXANet is 16.2 percentage points higher than that of YOLOv4-tiny, while the F1 value of YOLO-MXANet is 22.2 percentage points higher than that of YOLOv4-tiny.

**Figure 6:**
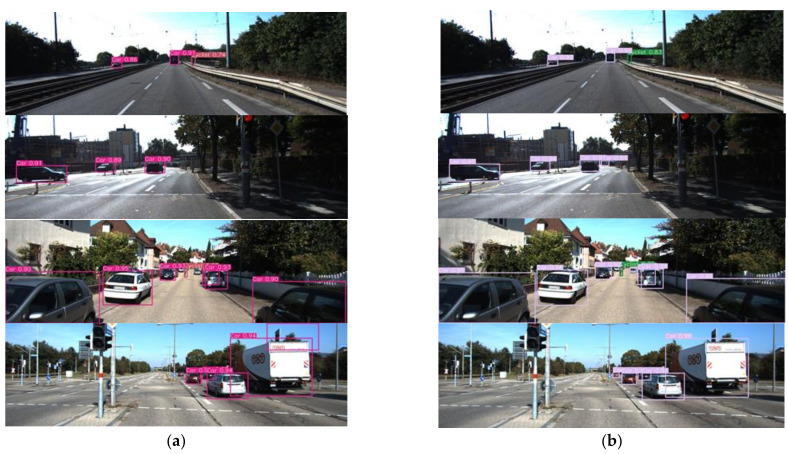
The detection results of YOLOv3 and YOLO-MXANet on the KITTI dataset. (**a**) YOLOv3. (**b**) YOLO-MXANet.

The actual detection results of YOLO-MXANet and YOLOv3 (baseline) on the KITTI dataset are compared in Figure 6. As can be seen from the comparison figures, YOLO-MXANet can detect complex objects that YOLOv3 cannot detect, such as small objects and occluded objects. Specifically, it can be seen from the first group of images that both YOLOv3 and YOLO-MXANet can detect three objects, but the confidence of bounding box of YOLO-MXANet is higher. As can be seen from the second group of images, YOLOv3 detects three objects “Car”, and YOLO-MXANet can detect four objects “Car”, in other words, YOLO-MXANet detects one smaller object with low light than YOLOv3, and the other three object’s bounding boxes detected by YOLO-MXANet have higher confidence. Similarly, it can be seen from the third group and the fourth group that YOLO-MXANet can also detect smaller objects and each bounding box detected by YOLO-MXANet has a higher confidence. This is because YOLO-MXANet can effectively enhance the characteristic information of object and suppress environmental interference.

### 3.3. Comparison Experiments with Other Algorithms on the CCTSDB Dataset

In order to further verify the performance of YOLO-MXANet, the comparative experiments with other advanced algorithms are conducted on the CCTSDB dataset. In the experiment, we select 3105 images from the CCTSDB dataset and use the dataset partition algorithm to randomly divide the CCTSDB dataset into train set and validation set and test set, and the 8:1:1 ratio is also adopted in this study. CCTSDB dataset is classified into three types of objects, namely, warning, prohibitory, and mandatory. The comparison results between YOLO-MXANet and the latest object detection algorithm on the CCTSDB dataset are shown in Table 3. Compared with YOLOv5m, YOLO-MXANet has fewer parameters and has more tremendous advantages in terms of detection accuracy. Compared with the lightweight algorithm YOLOv3-tiny, although the number of parameters of YOLO-MXANet is a little more than that of YOLOv3-tiny, the mAP 0.5 value of YOLO-MXANet is 6.8 percentage points higher than that of YOLOv3-tiny, and the F1 value of improved algorithm is 5.6 percentage points higher than that of YOLOv3-tiny. Compared with the lightweight algorithm YOLOv4-tiny, the mAP 0.5 value of YOLO-MXANet is 2.2 percentage points higher than that of YOLOv4-tiny, and the F1 value of improved algorithm is 7.7 percentage points higher than that of YOLOv4-tiny. Therefore, YOLO-MXANet is more suitable for object detection in traffic scenes.

**Figure 7:**
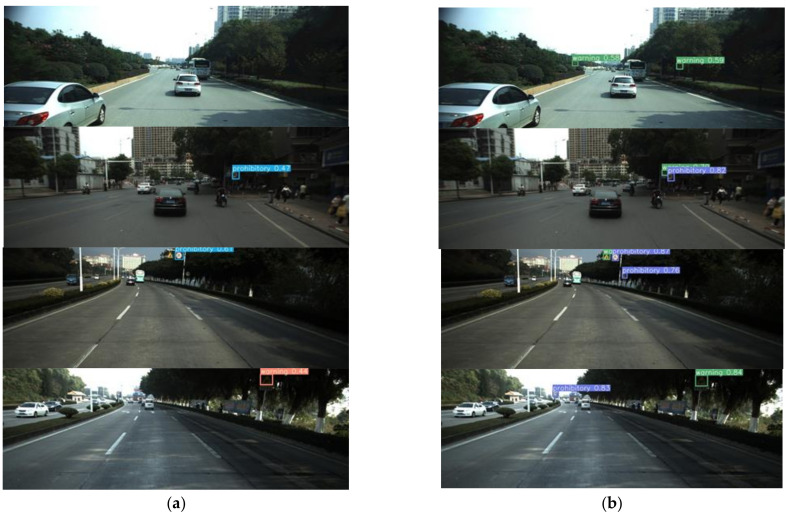
The detection results of YOLOv3 and YOLO-MXANet on the CCTSDB dataset. (**a**) YOLOv3. (**b**) YOLO-MXANet.

The comparisons of actual detection results between YOLO-MXANet and YOLOv3 (baseline) on the CCTSDB dataset are shown in Figure 7. In the first set of images, YOLO-MXANet can detect small and dim objects. As can be seen from the second group, YOLO-MXANet can detect the “warning” objects that YOLOv3 cannot detect, and the bounding box detected by YOLO-MXANet has a higher confidence. As we can see from the third pictures, YOLOv3 misses two objects, while YOLO-MXANet detects all of them. As can be seen from the fourth group of pictures, although YOLOv3 can detect large objects with dim light, the confidence of bounding box detected by YOLOv3 is not as high as that detected by YOLO-MXANet, and the detection ability of YOLOv3 is not as good as that of YOLO-MXANet in terms of small objects.

## 4. Conclusions

Based on general one-stage object detection algorithms, we propose a small object detection algorithm in traffic scenes (named YOLO-MXANet), which not only solves the problem that original algorithm is not high in detecting small-scale objects but also reduces the number of parameters from 61.5 M to 13.8 M and improves the detection speed. Therefore, YOLO-MXANet balances the detection accuracy, inference speed, and model complexity. We utilize CIoU to improve the loss function of YOLOv3 and improve the positioning accuracy of small objects. A lightweight backbone network (named MobileNeXt) is used to reduce the number of parameters and amount of computation, which can improve the detection speed of the model. However, the light weight will reduce the accuracy of the model to a certain extent. To further enhance the feature extraction capability of MobileNeXt, we present SA-MobileNeXt based on the Shuffle Channel and Spatial Attention module as the backbone network. In order to make up for the loss of precision caused by light weight, we use Mosaic and Mixup to train the model, which can enhance the ability of small object detection and thus improve the robustness of the model. To further enhance the characteristics of the small object, we add a Down-top path and fuse the four-fold subsampled feature maps from the backbone network. At the same time, to reduce the number of parameters without weakening the feature extraction ability of the network, we utilize the idea of CSPNet to combine convolution blocks. We perform ablation experiments on the KITTI dataset to demonstrate the effectiveness of each improved method. In addition, we conduct comparative experiments with other advanced algorithms on the KITTI and CCTSDB datasets, and the experimental results show that our algorithm has certain advantages in terms of detection accuracy, detection speed, and model complexity. Although our algorithm has achieved some improvements in accuracy and model complexity, there is still a long way to go before it can be deployed on mobile devices. Therefore, the next step is to further balance the detection accuracy, speed, and model complexity to provide excellent theoretical basis and practical value for intelligent transportation and unmanned driving.

## Figures and Tables

**Table 1:** The ablation experiments on the KITTI dataset.

Scheme	Method	P	R	F1	mAP 0.5	Speed_GPU_/ms	Params	GFLOPS
A	YOLOv3	0.923	0.765	0.837	0.888	3.5	61,508,200	154.9
B	A + CIoU	0.930	0.799	0.860	0.911	3.5	61,508,200	154.9
C	B + MobileNeXt	0.857	0.772	0.812	0.865	2.4	22,927,784	43.4
D	C + DA	0.882	0.806	0.842	0.897	2.4	22,927,784	43.4
E	D + PAN + 4s + BC	0.876	0.846	0.861	0.905	2.5	13,870,888	37.0
F	E + SiLU	0.941	0.822	0.877	0.916	2.5	13,870,888	37.0
G	F + A-MobileNeXt	0.943	0.818	0.876	0.922	2.9	13,987,271	37.1
H	G + SA-MobileNeXt	0.930	0.844	0.885	0.924	2.9	13,874,564	37.0

**Table 2:** The comparison results of the algorithms on the KITTI dataset.

Indicator	P	R	F1	mAP 0.5	Params (M)
Algorithm					
YOLOv3	0.923	0.765	0.837	0.888	61.5
YOLOv3-SPP	0.923	0.783	0.847	0.894	62.6
YOLOv5s	0.922	0.781	0.846	0.889	7
YOLOv5m	0.899	0.862	0.880	0.923	21.1
YOLOv3-tiny	0.763	0.598	0.670	0.692	8.7
YOLOv4-tiny	0.589	0.761	0.663	0.762	5.9
YOLO-MXANet	0.930	0.844	0.885	0.924	13.8

**Table 3:** The comparison results of the algorithms on the CCTSDB dataset.

Indicator	P	R	F1	mAP 0.5
Algorithm				
YOLOv3	0.910	0.894	0.902	0.928
YOLOv3-SPP	0.929	0.877	0.902	0.937
YOLOv5m	0.968	0.939	0.953	0.966
YOLOv3-tiny	0.911	0.873	0.892	0.905
YOLOv4-tiny	0.795	0.964	0.871	0.951
YOLO-MXAet	0.930	0.967	0.948	0.973

## Data Availability

Some or all data, models, or code generated or used during the study are available from the corresponding author by request.
